# Supplementary figures and images for: Exploring the interplay between the core microbiota, physicochemical factors, agrobiochemical cycles in the soil of the historic tokaj mád wine region
Source: PLoS One. 2024 Apr 16;19(4):e0300563. doi: 10.1371/journal.pone.0300563 (PMC11020696; doi:10.1371/journal.pone.0300563)

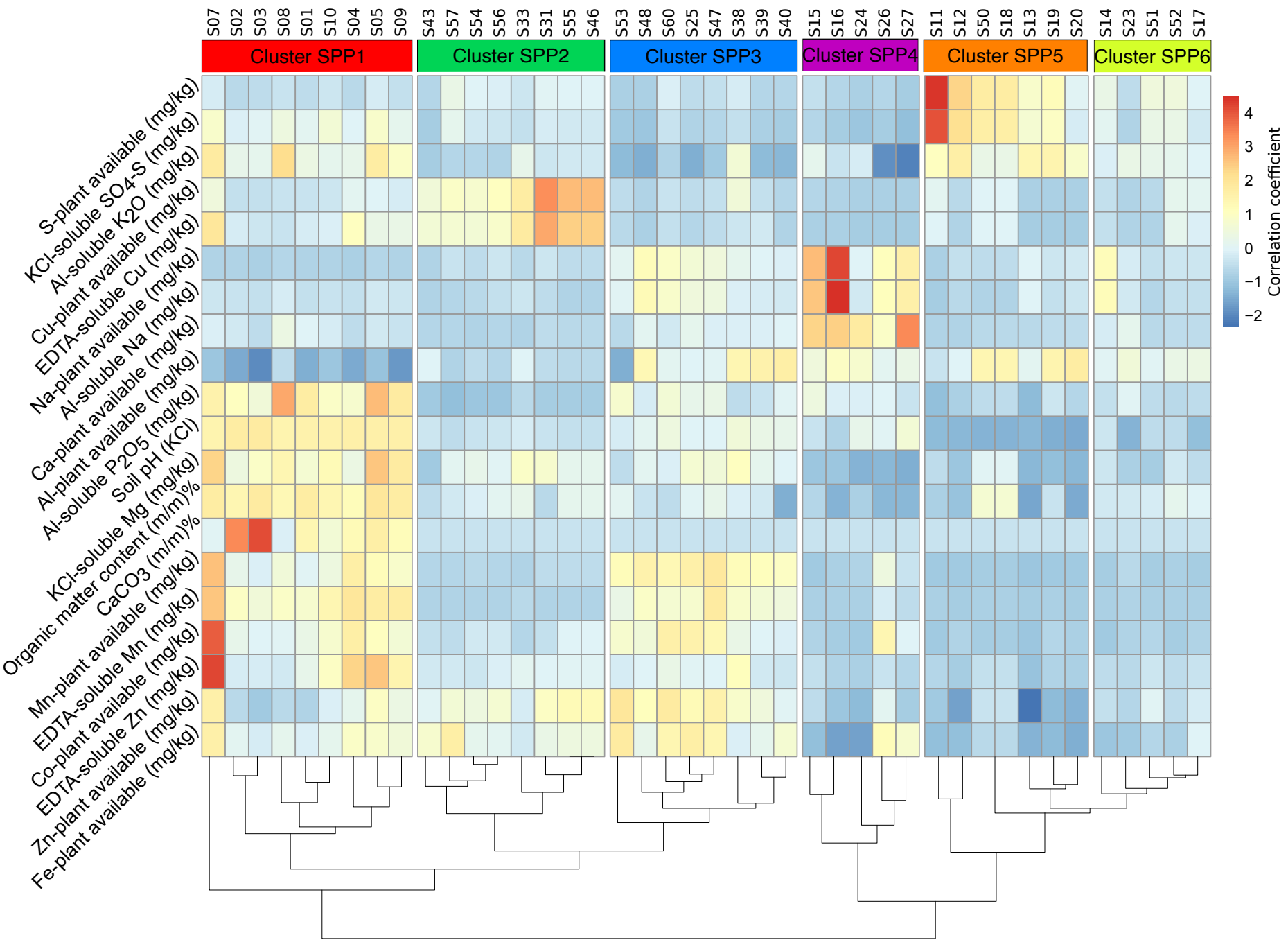

Supplement: S1 Fig — There were six distinct clusters identified according to the soil physicochemical properties (SPP): Cluster SPP1-Cluster SPP6. (PDF) [file pone.0300563.s001.pdf]
