# Supplementary material for: Exploring the interplay between the core microbiota, physicochemical factors, agrobiochemical cycles in the soil of the historic tokaj mád wine region
Source: PLoS One. 2024 Apr 16;19(4):e0300563. doi: 10.1371/journal.pone.0300563 (PMC11020696; doi:10.1371/journal.pone.0300563)
Supplement: S1 Table — The origin of each sample was specified, indicating the vineyard plot and soil depth. (DOCX) [file pone.0300563.s002.docx]

| **Sample**  **name** | **Vineyards** | **Sampling depth (cm)** | **Soil pH  (KCl)** | **CaCO_3_ (m/m)%** | **Organic matter (m/m)%** | **Al-soluble P_2_O_5_ (mg/kg)** | **Al-soluble K_2_O (mg/kg)** | **Al-soluble Na (mg/kg)** | **KCl-soluble Mg (mg/kg)** | **KCl-soluble SO_4_-S (mg/kg)** | **EDTA-soluble Mn (mg/kg)** |
| --- | --- | --- | --- | --- | --- | --- | --- | --- | --- | --- | --- |
| S1 | Király | 0-30 | 6.63 | 0.148 | 3.52 | 223 | 351 | 45.2 | 512 | 18.1 | 273 |
| S2 | Király | 0-30 | 6.86 | 0.202 | 3.18 | 197 | 332 | 46.2 | 431 | 15.5 | 296 |
| S3 | Király | 0-30 | 6.91 | 0.223 | 3.54 | 242 | 319 | 44.1 | 371 | 17.2 | 233 |
| S4 | Úrágya | 0-30 | 6.74 | 0.144 | 3.06 | 191 | 331 | 43.2 | 395 | 16.7 | 454 |
| S5 | Úrágya | 0-30 | 6.70 | 0.156 | 3.67 | 389 | 509 | 55.4 | 634 | 28.3 | 424 |
| S6 | Úrágya | 0-30 |  |  |  |  |  |  |  |  |  |
| S7 | Ősz-hegy | 0-30 | 6.57 | 0.112 | 3.51 | 371 | 519 | 51.8 | 492 | 28.6 | 559 |
| S8 | Danczka | 0-30 | 6.55 | 0.108 | 3.12 | 283 | 567 | 57.0 | 671 | 22.6 | 291 |
| S9 | Hold-völgy | 0-30 | 6.69 | 0.142 | 3.53 | 322 | 414 | 51.3 | 520 | 19.5 | 422 |
| S10 | Sarkad | 0-30 | 6.63 | 0.128 | 3.21 | 273 | 323 | 47.3 | 411 | 25.1 | 368 |
| S11 | Betsek | 0-30 | 3.74 | < 0.100 | 1.29 | 113 | 428 | 21.0 | 158 | 71.3 | 10.5 |
| S12 | Betsek | 30-60 | 3.66 | < 0.100 | 1.06 | 70.1 | 503 | 37.3 | 201 | 46.0 | 5.64 |
| S13 | Betsek | 60-90 | 3.61 | < 0.100 | 0.642 | 49.3 | 472 | 66.0 | 149 | 26.0 | 4.66 |
| S14 | Betsek | 0-30 | 4.68 | < 0.100 | 1.78 | 133 | 290 | 130 | 238 | 18.3 | 10.3 |
| S15 | Betsek | 30-60 | 4.56 | < 0.100 | 1.44 | 70.1 | 319 | 198 | 351 | 7.81 | 9.32 |
| S16 | Betsek | 60-90 | 4.19 | < 0.100 | 0.802 | 69.2 | 255 | 301 | 279 | 5.32 | 13.4 |
| S17 | Betsek | 0-30 | 3.78 | < 0.100 | 1.97 | 112 | 301 | 43.1 | 228 | 13.8 | 14.4 |
| S18 | Betsek | 0-30 | 3.59 | < 0.100 | 2.61 | 160 | 341 | 31.0 | 231 | 39.3 | 11.0 |
| S19 | Betsek | 30-60 | 3.49 | < 0.100 | 1.66 | 60.2 | 480 | 43.2 | 258 | 29.9 | 7.23 |
| S20 | Betsek | 60-90 | 3.43 | < 0.100 | 0.703 | 48.7 | 402 | 49.2 | 219 | 14.5 | 5.49 |
| S21 | Betsek | 0-30 |  |  |  |  |  |  |  |  |  |
| S22 | Betsek | 30-60 |  |  |  |  |  |  |  |  |  |
| S23 | Betsek | 0-30 | 3.57 | < 0.100 | 1.77 | 88.3 | 342 | 55.0 | 302 | 7.63 | 20.3 |
| S24 | Betsek | 30-60 | 5.28 | < 0.100 | 1.28 | 42.3 | 281 | 55.2 | 284 | 3.25 | 13.6 |
| S25 | Betsek | 0-30 | 4.95 | < 0.100 | 2.01 | 221 | 131 | 92.1 | 326 | 7.02 | 270 |
| S26 | Betsek | 30-60 | 5.04 | < 0.100 | 0.914 | 49.6 | 72.5 | 124 | 290 | 5.02 | 110 |
| S27 | Betsek | 60-90 | 5.66 | < 0.100 | 0.869 | 35.3 | 53.6 | 149 | 245 | 2.10 | 58.4 |
| S28 | Szilvás | 0-30 |  |  |  |  |  |  |  |  |  |
| S29 | Szilvás | 30-60 |  |  |  |  |  |  |  |  |  |
| S30 | Szilvás | 60-90 |  |  |  |  |  |  |  |  |  |
| S31 | Szent Tamás | 0-30 | 4.61 | < 0.100 | 1.49 | 230 | 265 | 40.0 | 188 | 14.3 | 23.9 |
| S32 | Szent Tamás | 60-90 |  |  |  |  |  |  |  |  |  |
| S33 | Szent Tamás | 0-30 | 4.83 | < 0.100 | 2.22 | 239 | 330 | 49.8 | 222 | 10.4 | 41.2 |
| S34 | Szent Tamás | 60-90 |  |  |  |  |  |  |  |  |  |
| S35 | Nyúlászó | 0-30 |  |  |  |  |  |  |  |  |  |
| S36 | Nyúlászó | 30-60 |  |  |  |  |  |  |  |  |  |
| S37 | Nyúlászó | 60-90 |  |  |  |  |  |  |  |  |  |
| S38 | Király | 0-30 | 5.59 | < 0.100 | 1.93 | 253 | 376 | 61.0 | 231 | 11.2 | 274 |
| S39 | Király | 30-60 | 5.34 | < 0.100 | 1.81 | 177 | 160 | 59.0 | 251 | 5.97 | 243 |
| S40 | Király | 60-90 | 5.25 | < 0.100 | 0.763 | 159 | 149 | 55.1 | 299 | 5.24 | 224 |
| S41 | Nyúlászó | 0-30 |  |  |  |  |  |  |  |  |  |
| S42 | Nyúlászó | 30-60 |  |  |  |  |  |  |  |  |  |
| S43 | Szent Tamás | 60-90 | 4.64 | < 0.100 | 1.55 | 78.3 | 196 | 27.6 | 181 | 5.48 | 20.8 |
| S44 | Szent Tamás | 60-90 |  |  |  |  |  |  |  |  |  |
| S45 | Szent Tamás | 0-30 |  |  |  |  |  |  |  |  |  |
| S46 | Szent Tamás | 0-30 | 4.54 | < 0.100 | 2.11 | 175 | 272 | 28.3 | 190 | 13.6 | 25.3 |
| S47 | Betsek | 0-30 | 4.96 | < 0.100 | 2.21 | 208 | 191 | 88.3 | 331 | 8.01 | 440 |
| S48 | Betsek | 30-60 | 5.11 | < 0.100 | 1.64 | 169 | 129 | 132 | 272 | 3.26 | 286 |
| S49 | Betsek | 60-90 |  |  |  |  |  |  |  |  |  |
| S50 | Betsek | 0-30 | 3.59 | < 0.100 | 2.61 | 160 | 341 | 31.0 | 231 | 39.3 | 11.0 |
| S51 | Betsek | 0-30 | 4.45 | < 0.100 | 2.11 | 138 | 326 | 43.0 | 222 | 21.0 | 12.5 |
| S52 | Betsek | 0-30 | 4.44 | < 0.100 | 1.85 | 81.7 | 331 | 31.3 | 231 | 20.9 | 10.2 |
| S53 | Betsek | 0-30 | 4.78 | < 0.100 | 2.09 | 116 | 155 | 69.9 | 391 | 4.02 | 192 |
| S54 | Szent Tamás | 0-30 | 4.57 | < 0.100 | 1.92 | 181 | 221 | 35.1 | 169 | 13.3 | 21.3 |
| S55 | Szent Tamás | 0-30 | 4.54 | < 0.100 | 2.11 | 175 | 272 | 28.3 | 190 | 13.6 | 25.3 |
| S56 | Szent Tamás | 0-30 | 4.63 | < 0.100 | 2.05 | 162 | 214 | 44.6 | 171 | 12.7 | 19.4 |
| S57 | Szent Tamás | 0-30 | 4.45 | < 0.100 | 1.81 | 169 | 219 | 33.5 | 155 | 19.3 | 20.3 |
| S58 | Király | 0-30 |  |  |  |  |  |  |  |  |  |
| S59 | Király | 0-30 |  |  |  |  |  |  |  |  |  |
| S60 | Király | 0-30 | 5.45 | < 0.100 | 1.56 | 149 | 211 | 110 | 381 | 11.6 | 261 |

| **Sample**  **name** | **EDTA-soluble**  **Zn (mg/kg)** | **EDTA-soluble Cu (mg/kg)** | **Al-plant available**  **(mg/kg)** | **Ca-plant available (mg/kg)** | **Co-plant available (mg/kg)** | **Cu-plant available (mg/kg)** | **Fe-plant available (mg/kg)** | **Mn-plant available (mg/kg)** | **Na-plant available (mg/kg)** | **S-plant available (mg/kg)** | **Zn-plant available (mg/kg)** |
| --- | --- | --- | --- | --- | --- | --- | --- | --- | --- | --- | --- |
| S1 | 4.77 | 10.6 | 16440 | 4750 | 8.70 | 20.9 | 16700 | 410 | 56.0 | 203 | 38.7 |
| S2 | 3.52 | 11.1 | 15986 | 3866 | 8.34 | 20.0 | 18100 | 480 | 57.9 | 172 | 40.0 |
| S3 | 3.57 | 9.57 | 12361 | 2681 | 6.61 | 18.5 | 16500 | 360 | 52.1 | 195 | 35.5 |
| S4 | 10.9 | 28.4 | 15750 | 2638 | 17.4 | 43.5 | 23500 | 970 | 50.0 | 187 | 50.3 |
| S5 | 11.5 | 17.5 | 18885 | 3834 | 13.5 | 38.0 | 23000 | 820 | 64.5 | 327 | 61.9 |
| S6 |  |  |  |  |  |  |  |  |  |  |  |
| S7 | 16.3 | 41.2 | 19220 | 4463 | 31.7 | 63.4 | 28267 | 1314 | 55.6 | 330 | 70.3 |
| S8 | 3.31 | 8.03 | 22640 | 6580 | 7.00 | 22.4 | 18400 | 590 | 66.7 | 250 | 40.6 |
| S9 | 8.25 | 17.6 | 13727 | 3089 | 11.0 | 32.0 | 21400 | 690 | 56.7 | 229 | 53.3 |
| S10 | 7.03 | 11.9 | 19059 | 4082 | 11.6 | 25.4 | 18700 | 540 | 53.3 | 281 | 46.5 |
| S11 | 2.11 | 12.8 | 23805 | 1711 | 1.02 | 42.7 | 10125 | 71.0 | 52.5 | 2124 | 35.6 |
| S12 | 1.58 | 3.33 | 26366 | 1812 | 2.35 | 11.6 | 10017 | 49.4 | 99.8 | 1325 | 26.2 |
| S13 | 0.901 | 1.46 | 27223 | 2311 | 0.647 | 4.54 | 8929 | 37.4 | 155 | 726 | 16.0 |
| S14 | 2.61 | 6.79 | 26663 | 4207 | 0.970 | 19.2 | 10953 | 92.8 | 331 | 534 | 41.7 |
| S15 | 2.69 | 2.59 | 30091 | 14986 | 2.08 | 8.82 | 10180 | 70.9 | 541 | 221 | 41.3 |
| S16 | 2.02 | 2.02 | 33681 | 15267 | 1.62 | 6.32 | 7118 | 112 | 755 | 143 | 34.0 |
| S17 | 2.92 | 12.2 | 28972 | 2668 | 2.33 | 42.9 | 17359 | 95.4 | 111 | 387 | 46.2 |
| S18 | 2.78 | 12.7 | 36533 | 2457 | 1.36 | 37.2 | 13796 | 91.2 | 90.1 | 1069 | 43.1 |
| S19 | 2.05 | 1.09 | 37015 | 2649 | 2.50 | 3.60 | 9540 | 62.1 | 119 | 867 | 32.0 |
| S20 | 1.78 | 0.845 | 38957 | 2719 | 1.67 | 2.81 | 8184 | 48.1 | 127 | 420 | 29.7 |
| S21 |  |  |  |  |  |  |  |  |  |  |  |
| S22 |  |  |  |  |  |  |  |  |  |  |  |
| S23 | 2.41 | 3.03 | 30599 | 5513 | 2.41 | 7.87 | 11592 | 178 | 134 | 202 | 39.0 |
| S24 | 1.78 | 1.17 | 31881 | 12739 | 1.10 | 4.45 | 7009 | 133 | 154 | 105 | 31.4 |
| S25 | 4.25 | 4.45 | 26059 | 5592 | 16.5 | 16.5 | 28492 | 950 | 248 | 204 | 65.6 |
| S26 | 2.88 | 1.18 | 27109 | 8809 | 15.5 | 3.50 | 25341 | 538 | 313 | 138 | 49.9 |
| S27 | 2.26 | 0.887 | 28750 | 19521 | 6.78 | 2.47 | 22636 | 253 | 382 | 63.7 | 35.9 |
| S28 |  |  |  |  |  |  |  |  |  |  |  |
| S29 |  |  |  |  |  |  |  |  |  |  |  |
| S30 |  |  |  |  |  |  |  |  |  |  |  |
| S31 | 3.95 | 56.2 | 22560 | 2695 | 3.98 | 190 | 19441 | 197 | 117 | 415 | 61.8 |
| S32 |  |  |  |  |  |  |  |  |  |  |  |
| S33 | 4.28 | 39.3 | 26271 | 1769 | 2.67 | 118 | 16063 | 143 | 75.8 | 273 | 45.4 |
| S34 |  |  |  |  |  |  |  |  |  |  |  |
| S35 |  |  |  |  |  |  |  |  |  |  |  |
| S36 |  |  |  |  |  |  |  |  |  |  |  |
| S37 |  |  |  |  |  |  |  |  |  |  |  |
| S38 | 7.27 | 9.04 | 36562 | 4367 | 8.97 | 64.6 | 16632 | 813 | 182 | 337 | 59.6 |
| S39 | 3.31 | 9.87 | 37530 | 4401 | 6.81 | 20.8 | 18761 | 787 | 156 | 163 | 55.5 |
| S40 | 3.18 | 3.48 | 38704 | 4804 | 6.19 | 10.7 | 22288 | 751 | 144 | 156 | 48.9 |
| S41 |  |  |  |  |  |  |  |  |  |  |  |
| S42 |  |  |  |  |  |  |  |  |  |  |  |
| S43 | 3.31 | 22.5 | 26240 | 2040 | 3.97 | 66.2 | 22537 | 162 | 56.5 | 159 | 48.8 |
| S44 |  |  |  |  |  |  |  |  |  |  |  |
| S45 |  |  |  |  |  |  |  |  |  |  |  |
| S46 | 4.09 | 48.3 | 21970 | 2438 | 6.47 | 163 | 20322 | 209 | 83.0 | 385 | 65.8 |
| S47 | 4.02 | 6.08 | 26757 | 4440 | 15.3 | 20.3 | 26576 | 1050 | 233 | 227 | 69.6 |
| S48 | 3.99 | 2.27 | 36060 | 5328 | 10.5 | 7.40 | 19216 | 927 | 332 | 87.3 | 64.0 |
| S49 |  |  |  |  |  |  |  |  |  |  |  |
| S50 | 2.78 | 12.7 | 36533 | 2457 | 1.36 | 37.2 | 13796 | 91.2 | 90.1 | 1069 | 43.1 |
| S51 | 2.55 | 15.5 | 28715 | 2739 | 1.62 | 48.1 | 12206 | 103 | 117 | 614 | 41.1 |
| S52 | 3.23 | 3.75 | 26676 | 2198 | 2.68 | 12.72 | 13057 | 87.5 | 94.4 | 610 | 48.6 |
| S53 | 3.51 | 5.02 | 16255 | 2242 | 11.9 | 16.4 | 29429 | 804 | 176 | 110 | 75.2 |
| S54 | 3.15 | 22.8 | 22740 | 1864 | 5.46 | 76.0 | 18742 | 174 | 90.9 | 380 | 54.6 |
| S55 | 4.09 | 48.3 | 21970 | 2438 | 6.47 | 163 | 20322 | 209 | 83.0 | 385 | 65.8 |
| S56 | 3.66 | 25.7 | 21774 | 2034 | 5.32 | 86.5 | 17958 | 162 | 115 | 359 | 58.3 |
| S57 | 3.41 | 23.9 | 21150 | 1904 | 3.48 | 79.8 | 28666 | 161 | 106 | 528 | 57.1 |
| S58 |  |  |  |  |  |  |  |  |  |  |  |
| S59 |  |  |  |  |  |  |  |  |  |  |  |
| S60 | 4.88 | 7.40 | 27179 | 3818 | 16.8 | 22.6 | 26580 | 863 | 293 | 353 | 73.3 |
