# Supplementary material for: Exploring the interplay between the core microbiota, physicochemical factors, agrobiochemical cycles in the soil of the historic tokaj mád wine region
Source: PLoS One. 2024 Apr 16;19(4):e0300563. doi: 10.1371/journal.pone.0300563 (PMC11020696; doi:10.1371/journal.pone.0300563)
Supplement: S2 Table — The nine different soil microbiome-associated ecosystem services were estimated based on the relative occurrence of microorganisms known to be involved in 1) improved soil aggregate formation, 2) production of antimicrobial agents, 3) siderophore production, 4) cellulose degradation, 5) production of antibiotics, 6) bioremediation, 7) nutrient mobilization, 8) plant growth stimulation, and 9) the production of phytohormone-like substances. The metabolic potential of these biological processes was classified into four evenly divided categories: 4-strong, 3-moderate, 2-weak, and 1-low. (DOCX) [file pone.0300563.s003.docx]

|  | **Improved soil aggregate formation** (11) | **Production of antimicrobial agents** (12) | **Siderophore production** (13, 14) | **Cellulose degradation** (15-17) | **Production of antibiotics** (18-20) | **Bioremediation** (21, 22) | **Nutrient mobilization** (23-26) | **Plant growth stimulation** (24, 25, 27-29) | **Production of phytohormone-like substances** (25) |
| --- | --- | --- | --- | --- | --- | --- | --- | --- | --- |
|  | *g_Lactobacillus* | *g_Penicillium* | *g_Pseudomonas* | *g_Pedobacter* | g_Pseudomonas | *g_Streptomyces* | *g_Azotobacter* | *g_Trichoderma* | *g_Agrobacterium* |
|  | *g_Pseudomonas* | *g_Aspergillus* | *s_Bacillus ssp.* | *g_Chryseobacterium* | g_Nocardia | *g_Clostridium* | *g_Flavobacterium* | *g_Alcaligenes* | *g_Rhizobium* |
|  | *g_Kocuria* |  | *s_B. polymyxa (Paenibacillus polymyxa)* | *g_Flavobacterium* | g_Streptomyces | *g_Rhodococcus* | *g_Bradyrhizobium* | *g_Agrobacterium* | *g_Bradyrhizobium* |
|  | *g_Azotobacter* |  | *g_Azospirillum* | *g_Arthrobacter* | g_Actinomadura | *g_Pseudomonas* | *g_Rhizobium* | *g_Azospirillum* | *g_Erwinia* |
|  | *g_Arthrobacter* |  | *s_Azotobacter vinelandii* | *g_Paenibacillus* | s_Micromonospora sp. |  | *g_Azospirillum* | *g_Arthrobacter* | *g_Azospirillum* |
|  | *g_Micrococcus* |  | *s_Bacillus subtilis* | *g_Streptomyces* | s_Bacillus ssp. |  | *g_Caballeronia* | *g_Herbaspirillum* | *g_Azotobacter* |
|  |  |  | *s_Pantoea allii* | *g_Agromyces* |  |  | *g_Burkholderia* | *g_Erwinia* | *g_Pseudomonas* |
|  |  |  | *s_Rhizobium radiobacter* | *g_Pseudomonas* |  |  | *g_Paraburkholderia* | *g_Enterobacter* | *s_Bacillus ssp.* |
|  |  |  |  | *g_Pseudoxanthomonas* |  |  | *g_Pseudomonas* | *g_Bradyrhizobium* |  |
|  |  |  |  | *s_Micrococcus* |  |  | *g_Sphingomonas* | *g_Rhizobium* |  |
|  |  |  |  | *g_Stenotrophomonas* |  |  | *s_Bacillus ssp.* | *g_Burkholderia* |  |
|  |  |  |  |  |  |  | *f_Methylobacteriaceae* | *g_Pseudomonas* |  |
|  |  |  |  |  |  |  | *f_Nitrosomonadaceae* | *g_Caulobacter* |  |
|  |  |  |  |  |  |  | *f_Nitrospiraceae* | *g_Chromobacterium* |  |
|  |  |  |  |  |  |  | *f_Rhodanobacteraceae* | *g_Flavobacterium* |  |
|  |  |  |  |  |  |  | *f_Rhodospirillaceae* | *g_Klebsiella* |  |
|  |  |  |  |  |  |  | *f_Alteromonadaceae* | *g_Mesorhizobium* |  |
|  |  |  |  |  |  |  | *f_Methylococcaceae* | *g_Micrococcus* |  |
|  |  |  |  |  |  |  | *f_Rhodobiaceae* | *g_Rhodococcus* |  |
|  |  |  |  |  |  |  |  | *g_Serratia* |  |
|  |  |  |  |  |  |  |  | *s_Lysinibacillus sphaericus* |  |
|  |  |  |  |  |  |  |  | *s_Bacillus amyloliquefaciens* |  |
|  |  |  |  |  |  |  |  | *s_Bacillus cereus* |  |
|  |  |  |  |  |  |  |  | *s_Bacillus mycoides* |  |
|  |  |  |  |  |  |  |  | *s_Bacillus subtilis* |  |
|  |  |  |  |  |  |  |  | *s_Bacillus pumilus* |  |
|  |  |  |  |  |  |  |  | *s_Bacillus thuringiensis* |  |
|  |  |  |  |  |  |  |  | *g_Azotobacter* |  |
|  |  |  |  |  |  |  |  | *s_Bacillus ssp.* |  |
|  |  |  |  |  |  |  |  | *g_Chaetomium* |  |
|  |  |  |  |  |  |  |  | *s_Botrytis cinerea* |  |
|  |  |  |  |  |  |  |  | *s_Sclerotinia sclerotiorum* |  |
|  |  |  |  |  |  |  |  | *s_Azotobacter vinelandii* |  |
|  |  |  |  |  |  |  |  | *B. polymyxa (Paenibacillus polymyxa)* |  |
| S1 | 1 | 1 | 1 | 2 | 2 | 2 | 3 | 2 | 1 |
| S2 | 1 | 1 | 1 | 1 | 2 | 2 | 3 | 2 | 2 |
| S3 | 1 | 1 | 1 | 1 | 1 | 1 | 4 | 2 | 1 |
| S4 | 1 | 1 | 1 | 1 | 1 | 1 | 4 | 2 | 2 |
| S5 | 1 | 1 | 1 | 2 | 2 | 2 | 3 | 2 | 1 |
| S6 | 1 | 1 | 1 | 2 | 2 | 2 | 3 | 2 | 2 |
| S7 | 1 | 1 | 1 | 2 | 2 | 2 | 4 | 2 | 2 |
| S8 | 1 | 1 | 1 | 2 | 2 | 2 | 3 | 2 | 2 |
| S9 | 1 | 1 | 1 | 2 | 2 | 2 | 3 | 2 | 2 |
| S10 | 1 | 1 | 1 | 2 | 2 | 2 | 3 | 3 | 2 |
| S11 | 1 | 4 | 1 | 2 | 3 | 3 | 2 | 2 | 2 |
| S12 | 1 | 2 | 1 | 2 | 3 | 2 | 2 | 3 | 2 |
| S13 | 1 | 3 | 1 | 2 | 2 | 2 | 2 | 3 | 3 |
| S14 | 1 | 3 | 1 | 2 | 2 | 2 | 2 | 3 | 2 |
| S15 | 1 | 3 | 1 | 2 | 2 | 2 | 2 | 3 | 2 |
| S16 | 2 | 1 | 1 | 3 | 3 | 3 | 1 | 2 | 1 |
| S17 | 1 | 1 | 1 | 2 | 2 | 2 | 2 | 3 | 3 |
| S18 | 1 | 2 | 1 | 2 | 2 | 2 | 2 | 3 | 3 |
| S19 | 1 | 2 | 1 | 2 | 3 | 2 | 2 | 3 | 2 |
| S20 | 1 | 1 | 1 | 2 | 3 | 3 | 1 | 3 | 2 |
| S21 | 1 | 4 | 1 | 3 | 3 | 3 | 2 | 2 | 2 |
| S22 | 1 | 1 | 1 | 2 | 3 | 3 | 1 | 2 | 2 |
| S23 | 4 | 1 | 1 | 4 | 2 | 2 | 1 | 3 | 1 |
| S24 | 3 | 1 | 1 | 4 | 2 | 3 | 1 | 3 | 1 |
| S25 | 1 | 1 | 1 | 3 | 4 | 3 | 1 | 1 | 1 |
| S26 | 2 | 1 | 1 | 3 | 2 | 2 | 1 | 3 | 2 |
| S27 | 2 | 1 | 1 | 4 | 3 | 3 | 1 | 2 | 1 |
| S28 | 1 | 1 | 1 | 3 | 3 | 3 | 2 | 2 | 2 |
| S29 | 1 | 1 | 1 | 3 | 3 | 3 | 1 | 2 | 2 |
| S30 | 1 | 1 | 1 | 4 | 3 | 3 | 1 | 2 | 1 |
| S31 | 1 | 1 | 1 | 4 | 4 | 4 | 1 | 1 | 1 |
| S32 | 2 | 1 | 2 | 2 | 2 | 2 | 1 | 4 | 2 |
| S33 | 1 | 1 | 1 | 3 | 3 | 3 | 2 | 2 | 2 |
| S34 | 1 | 1 | 1 | 1 | 1 | 1 | 2 | 4 | 4 |
| S35 | 1 | 3 | 1 | 4 | 4 | 4 | 1 | 1 | 1 |
| S36 | 1 | 1 | 1 | 4 | 4 | 4 | 1 | 1 | 1 |
| S37 | 4 | 1 | 1 | 4 | 3 | 3 | 1 | 3 | 1 |
| S38 | 1 | 1 | 1 | 4 | 3 | 3 | 1 | 2 | 1 |
| S39 | 1 | 1 | 1 | 4 | 3 | 3 | 1 | 2 | 1 |
| S40 | 1 | 1 | 1 | 3 | 3 | 3 | 2 | 2 | 2 |
| S41 | 1 | 2 | 1 | 3 | 4 | 4 | 1 | 2 | 2 |
| S42 | 1 | 1 | 1 | 1 | 2 | 2 | 2 | 4 | 3 |
| S43 | 1 | 1 | 1 | 3 | 4 | 4 | 1 | 1 | 1 |
| S44 | 1 | 1 | 1 | 1 | 1 | 1 | 2 | 4 | 4 |
| S45 | 1 | 1 | 1 | 1 | 1 | 1 | 3 | 3 | 3 |
| S46 | 1 | 1 | 1 | 4 | 4 | 4 | 1 | 1 | 1 |
| S47 | 1 | 1 | 1 | 3 | 3 | 3 | 2 | 2 | 1 |
| S48 | 1 | 1 | 1 | 3 | 3 | 3 | 1 | 2 | 2 |
| S49 | 1 | 1 | 1 | 4 | 4 | 4 | 1 | 1 | 1 |
| S50 | 1 | 1 | 1 | 1 | 2 | 2 | 2 | 4 | 3 |
| S51 | 1 | 1 | 1 | 1 | 1 | 1 | 2 | 4 | 3 |
| S52 | 1 | 1 | 1 | 1 | 1 | 1 | 2 | 4 | 3 |
| S53 | 2 | 1 | 3 | 2 | 2 | 2 | 2 | 3 | 2 |
| S54 | 2 | 1 | 2 | 3 | 2 | 2 | 1 | 3 | 2 |
| S55 | 2 | 1 | 1 | 2 | 1 | 1 | 2 | 4 | 2 |
| S56 | 1 | 1 | 1 | 2 | 2 | 2 | 2 | 4 | 3 |
| S57 | 1 | 1 | 1 | 1 | 1 | 1 | 4 | 2 | 1 |
| S58 | 1 | 1 | 2 | 2 | 1 | 2 | 2 | 4 | 2 |
| S59 | 2 | 1 | 4 | 3 | 2 | 2 | 1 | 2 | 2 |
| S60 | 3 | 1 | 4 | 2 | 2 | 3 | 1 | 1 | 2 |
