# Supplementary material for: Exploring the interplay between the core microbiota, physicochemical factors, agrobiochemical cycles in the soil of the historic tokaj mád wine region
Source: PLoS One. 2024 Apr 16;19(4):e0300563. doi: 10.1371/journal.pone.0300563 (PMC11020696; doi:10.1371/journal.pone.0300563)
Supplement: Attachment — Submitted filename: Review on manuscript.pdf [file pone.0300563.s004.pdf]

REVIEW OF MANUSCRIPT (PONE-D-23-21640) ENTITLED „EXPLORING THE INTERPLAY BETWEEN THE CORE MICROBIOTA, PHYSICOCHEMICAL FACTORS, AGROBIOCHEMICAL CYCLES AND RESISTOME IN THE SOIL OF THE HISTORIC TOKAJ MÁD WINE REGION" BY REMENYIK J. *ET AL*.

The soil microbial community plays a pivotal role in the growth and vigor of cultivated plant species, so the research aim is sensible and well-articulated. Soil samples, originated from ten vineyards of the Tokaj-Mád wine region were analyzed and compared based on a metagenomics research strategy. Characteristics of the soil microbial community were evaluated at three depths and at two-time points. The first question to come to light is related to the sampling strategy; authors indicated that altogether 60 soil samples were collected, nevertheless one sample consisted of 15-20 subsamples: **1)** *How the subsamples were pooled, soil or community DNA were combined?* **2)** *Why the percentages of top, middle and deep soil samples differ?* **3)** *What is the reason of soil sample number discrepancy mentioned in the text and S1 Fig. and Table (60 vs 42 respectively)?*

In order to correctly investigate the qualitative diversity and the community structures of the samples, rarefaction and/or rank abundance curves generation is critical. **4)** *Please demonstrate/ validate that the number of OTUs are saturated in all group samples to get enough sequencing depth.* **5)** *By the way, for some reason almost all of the figures and their labels are not sharp in the pdf version of the manuscript, however downloaded tiff files were legible.* **6)** *Please pay more attention to the proper preparation of the figure and table legends; do not use incorrect, inconsistent or unexplained abbreviations in the figures, or such that are not common knowledge.*

A few comments about the applied techniques and their presentation. The performed methods and techniques meet the current technological standards and are suitable for answering the scientific questions formulated in the objectives. Shotgun metagenomics is a powerful approach to assess microbial composition, diversity, and functional potential, **7)** *however, it is hard to believe that "direct analysis of individual reads rather than contigs", would provide more reliable results, especially in the case of short reads (2 × 151 bp).* **8)** *As for the quantity of reads generated from the samples – 20 million reads per sample – it highly questionable that such a uniform high-quality read count could be correct.* **9)** *Although it's a small thing, MagNA Lyser Instrument indicates oscillation frequency (speed) in revolutions per minute (rpm) not in G-force.* Nevertheless, these are minor remarks, not affecting unfavorably the value of the work presented. The results and discussion section nicely follows the logic of the materials and methods. It mostly presents the findings clearly. In general, the writing style of the manuscript is proper, and the text contains only a few typographic errors and ambiguous phrasing, however personally I would like to see a much more intense and detailed result comparison with previous knowledge during the discussion.
