# Supplementary material for: Exploring the interplay between the core microbiota, physicochemical factors, agrobiochemical cycles in the soil of the historic tokaj mád wine region
Source: PLoS One. 2024 Apr 16;19(4):e0300563. doi: 10.1371/journal.pone.0300563 (PMC11020696; doi:10.1371/journal.pone.0300563)
Supplement: Attachment — Submitted filename: PLoSONE Manuscript Review-Jan 2024.pdf [file pone.0300563.s007.pdf]

RESPONSE TO REVIEWER COMMENTS

Manuscript ID: PONE-D-23-2140R1

Manuscript Title: Exploring the interplay between the core microbiota, physicochemical factors, agrobiochemical cycles in the soil of the Historic Tokaj Mád Wine Region.

Authors: Judit Remenyik[1] & and László Csige[2] &, Péter Dávid[1], Péter Fauszt[1], Anna Anita Szilágyi-Rácz[1], Erzsébet Szőllősi[1], Zsófia Réka Bacsó[2], István Szepsy Jnr[2], Krisztina Molnár[4], Csaba Rácz[4], Gábor Fidler[1], Zoltán Kállai[3], László Stündl[5], Attila Csaba Dobos[4] and Melinda Paholcsek[1].

Confidential Comment to Editor:

Dear Editor, I strongly agree with the subject addressed in the manuscript and is worth investigation. Also, I agree that the information presented suites studies into the relationship between microbiota, physicochemical factors and agrobiochemical cycles in temperate regions and that the conclusions are supported by data. I also strongly agree that the manuscript is appropriate for the journal. The manuscript is appropriately organised. Further, I agree that the figures, tables and supplementary data are appropriate. The organisation of the manuscript is appropriate for PLoSONE journal. Please accept the manuscript for publication. The suggestion I pointed out were all addressed by the authors. This has improved the science and readability of the manuscript. My overall recommendation: Accept manuscript.

Reviewer: Date:

Johnny Kofi Awoonor 31st January, 2024

……………………………. ……………………………..
