# Supplementary material for: Exploring the interplay between the core microbiota, physicochemical factors, agrobiochemical cycles in the soil of the historic tokaj mád wine region
Source: PLoS One. 2024 Apr 16;19(4):e0300563. doi: 10.1371/journal.pone.0300563 (PMC11020696; doi:10.1371/journal.pone.0300563)
Supplement: Attachment — Submitted filename: Answers to reviewer_5.docx [file pone.0300563.s008.docx]

Dear Editor,

Below, you will find our responses to the questions raised by Reviewer 5. We appreciate the opportunity to address these inquiries and hope our answers clarify any concerns or issues highlighted by the reviewer.

Best regards,

Melinda Paholcsek

Answers to reviewer #5:

**L 147-148 “Principal coordinate analysis (PCoA) was performed to investigate clustering according to the soil physicochemical properties (Fig. 1C) and possible correspondence between the microbial taxonomy of the historical vineyards and the other (Fig. 1D).” Fig. 1C, Why the in the PCoA of soil properties most of the points are in the same coordinate of the axis 1 if the soils clearly present different features. Fig. 1D, It is not clear what is the point of the figure, or what are you showing.**

**In the case of Figure D, we would like to emphasize that, concerning the taxonomic composition of soils, the points representing the highlighted vineyards are closely situated, indicating a similarity based on taxonomy. However, compared to other vineyards, there are significant differences. With this, our goal is to demonstrate that the microbial fingerprints of the highlighted vineyards could be distinguished from those of the other areas. We also added a sentence to the discussion please see lines: 522-524. “Concerning the taxonomic composition of vineyard soils, it can be estimated that microbial fingerprints of the historic vineyards are distinguishable from those of other areas in the Tokaj-Mád wine region, a distinction that arises from the exhibited taxonomic similarities.”**

**The authors didn’t answer my question about the Figure 1C.**

Thank you for the question. In the case of Fig1C, the Principal Coordinate Analysis (PCoA) was performed, clustering according to the soil physicochemical properties. Here, a different dataset was used compared to Fig 1D, which is based on taxonomic data. The complexity of metagenomic datasets is higher than that of data related to soil physicochemical parameters. This difference in complexity might also contribute to the variations observed between the two figures, as metagenomic data encompass a broader spectrum of biological information that can influence the clustering patterns observed in the PCoA.

**L 155-157 “Remarkable intra-vineyard alterations were observed in the 20 most abundant fungal genera especially in the case of Szent Tamás, Betsek and Sarkad” Were are shown this results?**

**Yes, please see Fig1/F. Szent Tamás, Betsek and Sarkad Fungal compositions are remarkably different from other vineyards.**

**What you can see in figure 1f is the inter-vineyards variation and not intra-vineyards**

Thank you for noticing. The statement was changed to: Remarkable inter-vineyard alterations were observed in the 20 most abundant fungal genera especially in the case of Szent Tamás, Betsek and Sarkad. Please see line 168- 169

**L 299-302 “In the case of bacteria, spectacular differences in pattern dynamics were observed in community diversity, resulting in two clusters (postveraison – cluster 1, preharvest – cluster 2) with different spatial ordinations.” Did you did any statistical test support this conclusion?**

**No, this is based on the clusters in PCoA plot.**

**In PCoA plot to define valid cluster a statistical test should be done**

Thank you for the question. The ANOSIM statistical test was employed to calculate the statistics for the Principal Coordinate Analysis (PCoA). As indicated on lines 340-341, for bacteria, distances revealed two clusters with significantly different taxonomic compositions (p = 0.01), whereas fungi displayed overlapping clusters, suggesting more similar taxonomic compositions (p = 0.165).

Furthermore, we have included a statement in the materials and methods section under Statistical Analysis, with a reference, as seen on lines 686-688: "For the statistical analysis of Principal Coordinate Analysis (PCoA), the ANOSIM permutation test was utilized to evaluate the statistical significance of the separation among groups, using the R 'vegan' package (57)."

For reference, please see lines 893-895: Cho EJ, Leem S, Kim SA, Yang J, Lee YB, Kim SS, et al. "Circulating Microbiota-Based Metagenomic Signature for Detection of Hepatocellular Carcinoma." Sci Rep. 2019 May 17;9(1):7536.
