# Supplementary material for: Exploring the interplay between the core microbiota, physicochemical factors, agrobiochemical cycles in the soil of the historic tokaj mád wine region
Source: PLoS One. 2024 Apr 16;19(4):e0300563. doi: 10.1371/journal.pone.0300563 (PMC11020696; doi:10.1371/journal.pone.0300563)
Supplement: Attachment — Submitted filename: PLoSONE PONE-D-23-21640 Reviewer Comment - November 2023.pdf [file pone.0300563.s005.pdf]

Reviewer Comment for Manuscript PONE-D-23-21640: Exploring the Interplay Between the Core Microbiota, Physicochemical Factors, Agrobiochemical Cycles and Resistome in the Soil of the Historic Tokaj Mád Wine Region

Summary of the studies:

Shotgun metabarcoding was used in the assessment of microbial-terroir in the Tokaj-Mád vineyards of Hungary. The mutual interactions between soil diversity, functional potential and soil chemistry were investigated across the 0 - 90 cm soil layers (0 - 30 cm, 31 - 60 cm and 61 - 90 cm). The results from 60 samples indicated that *fungal phyla, Ascomycota, Basidiomycota,* and *Mucoromycota* were operating at 97% operational taxonomic units. By investigating the correlation between the 26 biological activities and the soil physico-chemical parameters, the authors found that soil Na (both plant available and non-available), similar to K_2O and P_2O_5 contents, stimulated most of the agrobiochemical cycles studied, except linoleic acid metabolism, alkaloid and anthocyanin biosynthesis. Soil bacterial diversity was positively correlated with the vast majority of the examined soil physico-chemical characteristics, while fungal diversity was negatively correlated with these characteristics. The pH and $CaCO_3$ content were inversely proportional to the 26 agrobiochemical cycles investigated with the exception that highly calcareous (i.e., alkaline) soils favoured the biosynthesis of alkaloids and isoflavonoids. From the discussion, the natural conditions, altitude, bedrock, micro- and mezzo-climatic factors, and concentration of trace elements in the soil render the Mád wine region suitable for making single-vineyard wines, thus contributing to some of their distinctive characteristics and superb quality. The authors observed that temporal factors exerted a marked influence on soil bacterial community composition. Fungal compositions showed a higher correspondence due to ripening confirms the general observation that community resilience increases with diversity. This also suggests that diverse soil fungal symbiomes are more resistant to external interventions. The authors conclude that, understanding the contribution of soil microbial communities to soil agrobiochemical cycles is critical for predicting terrestrial ecosystem feedbacks.

General Remarks:

This is an interesting and well written manuscript. The title is appropriate for the content of this article. The abstract is concise and accurately summarizes the essential information of the paper. The introduction provides an excellent, generalized background of the topic and this gives the reader an appreciation of the wide range of applications to the technology (nucleic acid extraction and shotgun sequencing) used for this research. The authors collected a unique dataset and the data were analyzed following laid down scientific methodology. The experimental design is standard and appropriate for this study. The authors provided adequate information especially in the materials and methods to make this research experiment capable of been reproduced by a capable research scientist.

The main strength of this paper is that it addresses an interesting topic and it contributes to our understanding on the importance of soil resistome studies and the associated antibiotic resistance genes (ARG) dissemination mechanisms. The manuscript also finds a novel solution based on a carefully selected set of rules and procedures and finally provides a clear solution to the research

questions asked. Literature cited in the manuscript, are relevant for this study. The figures and tables are well organized, easy to interpret and follows a systematic and a logical order. It improves our understanding of the importance of the spatial variability of soil resistomes and how ARG depends on microbial community structure as well as its functional potential.

Generally, the study is of great significance and contributes extensively to our understanding of soil microbial communities to soil agrobiochemical cycles is critical for predicting terrestrial ecosystem feedbacks in the study area. I recommend this manuscript (PONE-D-23-21640) for publication after some corrections have been taken into account. Thus, to make the manuscript more reader friendly to an international audience, I suggest that the authors, if possible, should rephrase the title of the manuscript, some sentences in the manuscript and also include the significant level (e.g., 0.05, 0.01, 0.001) and the p - values (e.g., $p < 0.03$), if possible, into their findings where significant or not significant and/or where positive or negative corelation ($r = 0.00$; p -value) is reported in the manuscript.

Specific Remarks:

Pg. 3
Line 83 - 84: Metabolites produced by the rhizobiome are important contributors to the quality, fragrance and distinctive personality of the products (5,6).

Please replace *"personality"* with *"quality".*

Pg. 4
Line 86 - 88: The adaptation abilities of the microbiota depend on community plasticity. Network analysis-based approaches can help in better understanding the intricate nature of microbe–microbe and microbe-community interactions.

Please rephrase the above sentence as: *The adaptation abilities of the microbiota depend on community plasticity. Network analysis-based approaches can help understand the intricate nature of microbe–microbe and microbe-community interactions.*

Pg. 10
Line 244 - 245: At the phylum level, 90.0% of the *Bacteria* and 70.0% of the *Fungi* OTUs were present in our sample population.

Please edit as: *"At the phylum level, 70% and 90% of Fungi OTUs and Bacteria were present in our sample population, respectively".*

Pg. 11
Line 255 - 256: Comprehensive comparisons of the soil samples of the Tokaj-Mád vineyards were made based on the hierarchical clustering of the bacterial and fungal 100% core microbiota (**Fig. 5**).

Please rephrase the above sentence as: *Comprehensive comparisons of the soil samples of the Tokaj-Mád vineyards were made based on the hierarchical clustering of the bacterial and fungal's 100% core microbiota (**Fig. 5**).*

Pg. 12
Line 279 - 280: *Verrucomicrobia* (0.066% - 9.25%)

Please rewrite as: *"Verrucomicrobia (0.66% - 9.25%)"*.

Pg. 14
Line 321: **Fig. 6:** Soil bacterial community changed significantly due to ripening.

Suggestion: *Please do insert the significant level (e.g., p-value = 0.05, or 0.01, or 0.001) and if possible, the significant value (e.g., 0.03).*

Pg. 14
Line 326 - 328: Based on an investigation of the soil core, the bacterial alpha diversity was significantly lower in the preharvest samples than in the postveraison samples.

Please insert the significant level and the p-value after the significantly (eg, $p < 0.05$, $p = 0.03$). The authors should take not and insert the significant values ($p = 0.03$) and the significant level ($p < 0.05$) in their script. This will enrich the findings of this research as well as prevent readers from searching for these in the tables and figures.

Pg. 17
Line 397: Microbial biological activities such as nitrogen

Please rewrite as: "*Microbiological activities such as nitrogen…………….*"

Pg. 18
Line 433: …..the levels were significantly higher only in relation to the lowest layer (61-90 cm).

Please insert the significant level and/or the p-value.

Pg. 21
Line 513-514: In Mád, a relatively dry August and September is conducive to grapes achieving full biological ripeness before the onset of overripening before the berries begin to shrivel.

Please rephrase as: "*In Mád the relatively dry climate in August and September is conducive for grapes achieving full biological ripeness before the onset of overripening and before the berries begin to shrivel*"

Pg. 22
Line 525-526: *Rhizopodaceae* and *Mycosphaerellaceae* have been found to become more influential throughout the ripening period.

Rewrite as: "*Rhizopodaceae and Mycosphaerellaceae have been found to be more influential throughout the ripening period*"

Figures and Tables

Dear authors, please increase the resolution of the figures and tables. For example, Figures 2A, and 2B, Figures 7A, and 7B, Figures 9D, and 9F.

Thank you.

Johnny Kofi Awoonor
(Research Scientist: CSIR-Soil Research Institute, Kumasi. Ghana)
